# [Retracted] Long non‑coding RNA SNHG6 promotes tumorigenesis in melanoma cells via the microRNA‑101‑3p/RAP2B axis

**DOI:** 10.3892/ol.2024.14274

**Published:** 2024-02-05

**Authors:** Hong Zhou, Lingqiao Li, Yingqian Wang, Dewei Wang

Oncol Lett 20: 323, 2020; DOI: 10.3892/ol.2020.12186

Following the publication of this paper, it was drawn to the Editor's attention by a concerned reader that certain of the colony formation assay data shown in Fig. 2C were strikingly similar to data appearing in different form in other research articles written by different authors at different research institutes that had either already been published, or were submitted for publication at around the same time.

Owing to the fact that contentious data in the above article had already been published elsewhere prior to its submission to *Oncology Letters*, the Editor has decided that this paper should be retracted from the Journal. The authors were asked for an explanation to account for these concerns, but the Editorial Office did not receive a reply. The Editor apologizes to the readership for any inconvenience caused.

